# Abnormalities in spinal cord ultrastructure in a rat model of post-traumatic syringomyelia

**DOI:** 10.1186/s12987-020-0171-4

**Published:** 2020-02-29

**Authors:** Joel Berliner, Sarah Hemley, Elmira Najafi, Lynne Bilston, Marcus Stoodley, Magdalena Lam

**Affiliations:** 1 Faculty of Medicine and Health Sciences, 2 Technology Place, Macquarie University, Sydney, NSW 2109 Australia; 2 Neuroscience Research Australia, Margarete Ainsworth Building, 139 Barker Street, Randwick, NSW 2031 Australia; 3 Prince of Wales Clinical School, Faculty of Medicine, University of New South Wales, Randwick, NSW 2031 Australia

**Keywords:** Post-traumatic syringomyelia, Cerebrospinal fluid, Spinal cord, Perivascular space, Ultrastructure

## Abstract

**Background:**

Syringomyelia is a serious complication of spinal cord trauma, occurring in approximately 28% of spinal cord injuries. Treatment options are limited and often produce unsatisfactory results. Post-traumatic syringomyelia (PTS) is presumably related to abnormalities of cerebrospinal fluid (CSF) and interstitial fluid hydrodynamics, but the exact mechanisms are unknown.

**Methods:**

Transmission electron microscopy (TEM) was used to investigate in detail the interfaces between fluid and tissue in the spinal cords of healthy Sprague–Dawley rats (n = 3) and in a rat model of PTS (n = 3). PTS was induced by computer-controlled impact (75 kDyn) to the spinal cord between C6 and C8, followed by a subarachnoid injection of kaolin to produce focal arachnoiditis. Control animals received a laminectomy only to C6 and C7 vertebrae. Animals were sacrificed 12 weeks post-surgery, and spinal cords were prepared for TEM. Ultra-thin spinal cord sections at the level of the injury were counterstained for structural anatomy.

**Results:**

Spinal cords from animals with PTS displayed several abnormalities including enlarged perivascular spaces, extracellular edema, cell death and loss of tissue integrity. Additionally, alterations to endothelial tight junctions and an abundance of pinocytotic vesicles, in tissue adjacent to syrinx, suggested perturbations to blood-spinal cord barrier (BSCB) function.

**Conclusions:**

These findings support the hypothesis that perivascular spaces are important pathways for CSF flow into and out of the spinal cord, but also suggest that fluid may enter the cord through vesicular transport and an altered BSCB.

## Background

From months to decades after a spinal cord trauma, PTS may develop within spinal cord tissue [1–3]. The fluid-filled cavities (syrinxes) that develop in PTS can enlarge over time and alter surrounding nervous tissue integrity. Damage to cord tissue can result in pain and neurological deficits. Treatment options for syringomyelia are limited to surgical decompression or correction of deformity, arachnolysis or shunting procedures to facilitate drainage [4–6]. Surgical treatments are invasive, carry high risks of complication, and result in unsatisfactory long-term success rates in approximately 50% of cases [2]. An improved understanding of the neuropathology of PTS is necessary to mitigate poor treatment outcomes.

The mechanisms leading to syrinx formation post-trauma are poorly understood. It is thought that formation and enlargement of the syrinx are related to abnormalities of CSF hydrodynamics, although the mechanism and route of fluid entry are unclear. Early theories proposed that CSF enters the cord from the 4th ventricle [7, 8], but it is now recognized that in most cases there is no direct communication between the syrinx and the 4th ventricle. CSF flow obstruction in the spinal subarachnoid space has been suggested to increase pressure and thus force fluid into the cord [9–11]. Indeed, in a recent rodent study, a spinal subarachnoid space obstruction increased CSF tracer penetration in spinal cord tissue [12]. This finding aligns with the observation that most post-traumatic syrinxes are associated with narrowing or obstruction of the spinal subarachnoid space [2]. An imbalance between fluid inflow from, and fluid outflow to, the spinal subarachnoid space may result in fluid accumulation within the cord—a state that presumably precedes syrinx formation [13]. Intraoperative observations indicate that fluid inside the syrinx is under high pressure. However, the existing theories on syrinx enlargement do not adequately explain how fluid flow continues against a pressure gradient.

There is a paucity of studies investigating the ultrastructure of the spinal cord in PTS. In ultrastructure studies on communicating syringomyelia (where the syrinx communicates with the fourth ventricle), enlargement of the central canal and concomitant hydrocephalus were induced experimentally by an intracisternal injection of kaolin [14, 15]. In the early stage of syrinx formation (2–6 weeks), demyelination and edema in the white matter adjacent to the syrinx coincided with astrocytic proliferation of the syrinx border. Rupture of these syrinxes was generally directed dorsolaterally [14]. Ultrastructural changes were consistent in the late stage of syrinx formation (6–12 months), however edema was less prevalent and astrogliosis more severe [15]. Enlarged perivascular spaces were found throughout the parenchyma at both stages of syrinx formation [14, 15], which suggests a pathological role. This model, however, does not describe the ultrastructural changes that occur in non-communicating syringomyelia.

Perivascular spaces are known to provide a major pathway for fluid flow into the cord [16–18] and are hypothesized as the main path for fluid and waste clearance [19]. In rodent models of syringomyelia, rapid influx of tracer from spinal subarachnoid space to perivascular spaces was reported [3, 20]. However, the contribution of perivascular flow to syrinx formation is unclear. A recent electron microscopic study in healthy rats indicated a continuity of flow between the spinal subarachnoid space and the central canal, via a network of perivascular and extracellular spaces in the white and grey matter [21]. Whether the ultrastructure of perivascular spaces and fluid interfaces of the spinal cord is altered in PTS is yet to be determined. The present study used TEM to examine spinal cord ultrastructure in a rat model of PTS, with particular focus on perivascular spaces and tissue adjacent to syrinx cavities.

## Methods

All experimental methods involving animals were performed according to the Australian Code for the Care and Use of Animals for Scientific Purposes, and were approved by the Macquarie University Animal Ethics Committee (ARA 2016/032). Experimental procedures were carried out on six male Sprague–Dawley rats that weighed 424 ± 23 g (mean ± SD) and were aged between 9 and 12 weeks. In a sterile field, general anesthesia was induced with 5% isoflurane in oxygen (1 L/min) and maintained at 2–2.5% isoflurane in oxygen (0.2 L/min), adjusted according to physiological parameters. PTS was induced in three rats as previously described [22]. Briefly, a computer-controlled Infinite Horizon Impactor (Precision Systems and Instrumentation, LLC, Kentucky, USA) was used to produce an initial injury at spinal segments C6–C8 with a force of 75 kDyn, followed by a subarachnoid injection of kaolin at the site of injury to produce a focal obstruction and arachnoiditis. Arachnoid adhesions and obstruction to the spinal subarachnoid space are commonly observed at the level of the syrinx in PTS patients, and are thought to contribute to syrinx formation and/or enlargement [23–25]. Subsequent hemorrhage of the dorsal vein was an indicator of a successful impact and injury to the cord. Apart from occasional hemorrhage of small vessels feeding the dorsal vein, other surface vasculature remained unaffected by the impact. The combination of injury and arachnoiditis with subarachnoid space obstruction produces an extracanalicular cavity that enlarges slightly over time [26]. Control animals (n = 3) had laminectomy surgery only. The surgical site was closed with 4–0 Absorbable Coated Vicryl sutures (Ethicon, Johnson & Johnson Medical Pacific Pty Ltd, Sydney, Australia). After the operations, 0.05 mg/kg of 300 µg/mL buprenorphine was administered subcutaneously. Animals were closely monitored for any signs of excessive weight loss, limb weakness, urinary retention or excessive self-grooming. Subsequent doses of buprenorphine were given until alleviation of post-surgery deficits. Food and water were allowed ad libitum and saline was administered subcutaneously for animals that appeared dehydrated.

Twelve weeks after the induction of syringomyelia, to allow sufficient time for the development of an enlarged syrinx, animals were placed under general anesthesia (5% isoflurane in oxygen induction and maintained at 2.5% isoflurane in oxygen). Animals were intracardially perfused with a mixture of paraformaldehyde (3%) and glutaraldehyde (2.5%) in sodium phosphate buffer (0.1 M), and the spinal cord dissected out and processed for electron microscopy, as described previously [21]. In short, 1 mm thick spinal cord segments underwent post-fixation and en bloc staining with osmium tetroxide (1%; 1 h) and uranyl acetate (2%; 30 min) respectively, followed by ethanol dehydration in graded solutions and LR White resin infiltration (ProSciTech, Queensland, Australia). Blocks of polymerized resin were sectioned using a Leica EM UC7 ultramicrotome (Wetzlar, Germany). Orientation of tissue sections was established through semi-thin sections (750 nm) stained with a solution of methylene blue (1%), sodium bicarbonate (0.6%) and glycerol (40%). Ultra-thin sections (70–80 nm) were mounted and dried on coated copper grids. Sections were stained on-grid with uranyl acetate (7%; 8 min) and Reynolds lead citrate (3 min). Imaging was carried out using a Philips CM10 TEM equipped with a Mega view G2 digital camera (Olympus SIS, Münster, Germany). The ultrastructure of the perivascular spaces in spinal cord tissue in direct contact with the syrinx cavity, as well as rostral and caudal to the syrinx, were examined in at least three sections from three separate spinal cord levels in each animal.

## Results

The TEM analyses of multiple transverse sections from lower cervical and upper thoracic spinal cord segments revealed several anatomical abnormalities in this PTS model, discussed in detail below. Perivascular spaces were defined by largely electron-lucent spaces between the vascular wall and the foot processes of astrocytes in the parenchyma. These spaces were often filled with collagen fibrils. The thinning of these spaces at the level of capillaries leaves only the basal lamina, a layer of extracellular matrix and collagen between a single layer of endothelium and the plasma membrane of parenchymal cells, most typically astrocytes. The extracellular space was described as the space between parenchymal cells (astrocytes, oligodendrocytes, neurons and their processes) separated from the CSF-filled areas of the subarachnoid and perivascular space by the foot processes of astrocytes. These fluid compartments are not truly separate, as gap junctions exist between astrocytic foot processes that allow extracellular fluid to communicate with perivascular spaces and basal laminae [21].

### Inflammatory processes in PTS model

**Fig. 1:**
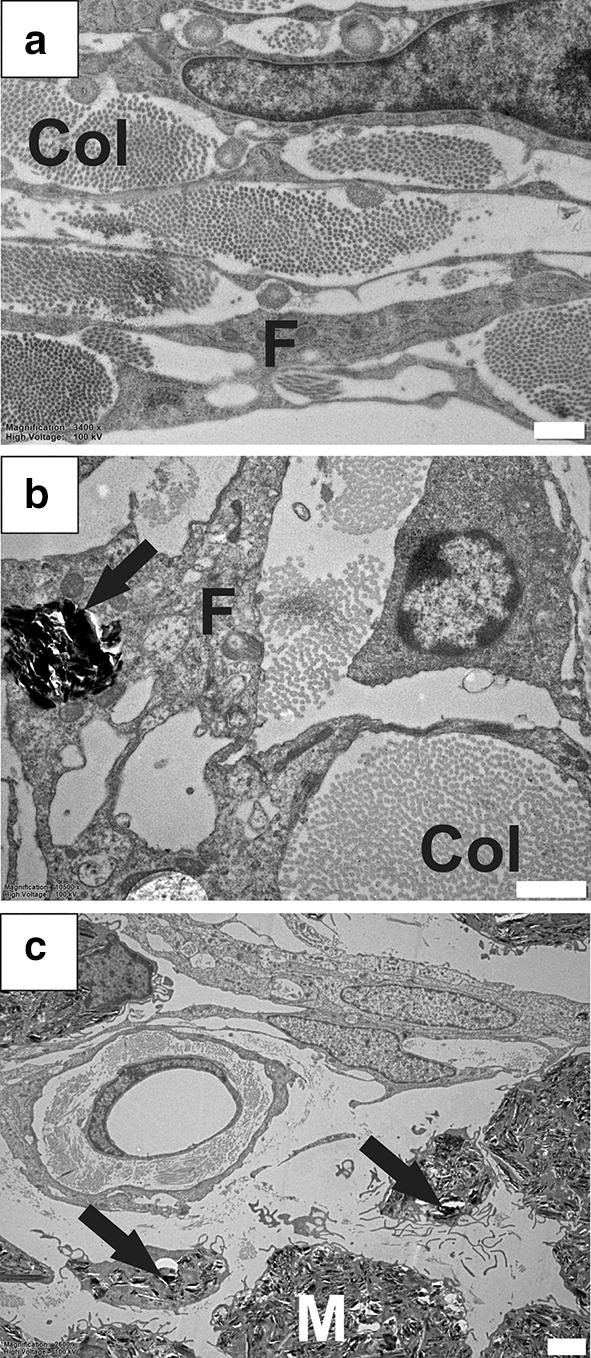
Inflammatory response in the spinal subarachnoid space. Typical subarachnoid space appearance in healthy animals (**a)** containing cellular processes of fibrocytes, and abundant collagen fibres. In a rat model of PTS, intracellular kaolin deposits (black arrows) localize to subarachnoid space fibrocytes (**b**) and macrophages (**c**). Col, collagen; F, fibrocytes; M, macrophage. Magnification: ×3400 (**a**), ×10,500 (**b**), ×2600 (**c**). Scale bars: 2 µm (**a**, **c**), 1 µm (**b**)

A strong inflammatory response to kaolin was observed primarily in the spinal subarachnoid space (Fig. 1). Inflammation in the subarachnoid space was evidenced by numerous kaolin-filled phagocytic cell bodies (Fig. 1b, c). Some of these cells were present at the spinal nerve rootlets. Inflammatory cells were observed in the white and grey matter including cells that were different in appearance to the kaolin-induced macrophages, and resembled activated macrophages.

### Enlarged perivascular spaces

**Fig. 2:**
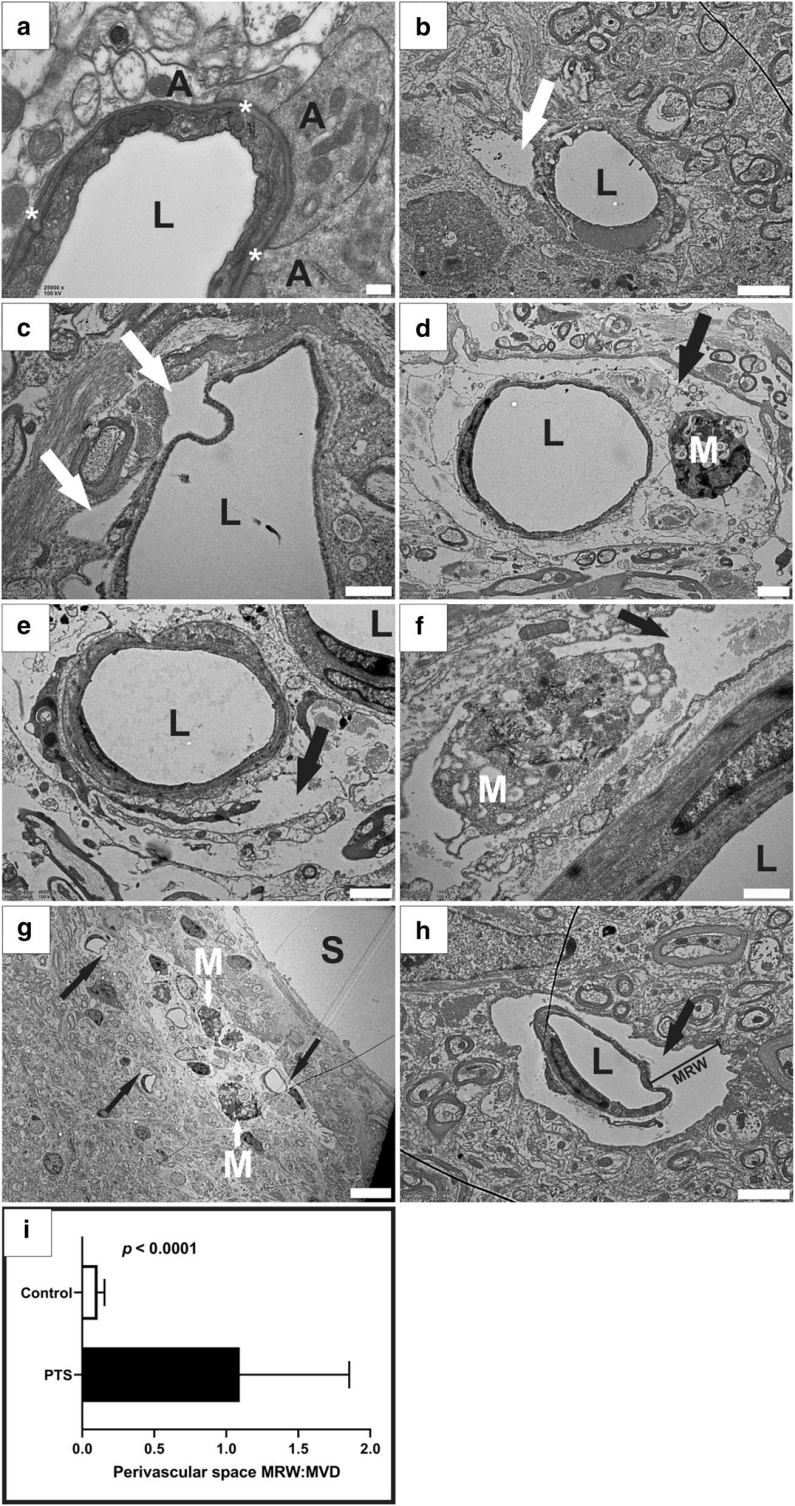
Enlarged perivascular spaces in animals with PTS. Normal appearing blood vessel in the spinal cord from a healthy control animal (**a**). Perivascular microcavities were identified in some blood vessels in PTS animals (white arrows) and were hypothesized as an early stage pathological process that leads to enlarged perivascular spaces (**b**, **c**). Dilated perivascular spaces were observed in the vicinity of cavities around all types of blood vessels in PTS animals; examples of enlarged perivascular spaces (black arrows) around venules (**d**), arterioles (**e**, **f**) and capillaries (**g**, **h**). A maximum radial width (MRW) measurement is shown around an enlarged perivascular space of a capillary (**h**). The ratio of MRW of the perivascular space to maximum vessel diameter (MVD) was significantly larger in PTS animals (n = 11) when compared to controls (n = 15): unpaired t-test, *p* < 0.0001 (**i**). A, astrocyte; L, lumen; M, macrophage; S, syrinx; *, basal laminae. Magnification: ×25,000 (**a**), ×5800 (**b**, **h**), ×10,500 (**c**, **f**), ×3400 (**d**), ×4600 (**e**), ×1450 (**g**). Scale bars: 0.2  µm (**a**), 2  µm (**b**, **d**, **e**, **h**), 1 µm (**c**, **f**), 5  µm (**g**)

Perivascular spaces of arterioles and venules in the healthy spinal cord ranged from 264 nm to 1.9 µm in radial width, in line with previous data [21]. At the level of capillaries in healthy spinal cord, the perivascular space was reduced to a thin (50–100 nm) layer of electron-dense extracellular matrix, the basal lamina (Fig. 2a, see asterisks). In rats with PTS, some blood vessels exhibited either perivascular microcavities that affected only a small section of its perimeter (Fig. 2b, c), or the entire perivascular space was significantly enlarged with partial or complete loss of the connective tissue (Fig. 2d–h). Enlarged perivascular spaces were observed around all types of blood vessels including venules (Fig. 2c, d), arterioles (Fig. 2e, f), and capillaries (Fig. 2g, h). In PTS animals, maximum radial width (MRW) of the perivascular space from the vessel wall ranged from 2.4 to 30.2 µm for venules, 2.1 to 14.8 µm for arterioles, and 954 nm to 4.1 µm for capillaries. Irrespective of vessel type, the MRW of the perivascular space as a ratio of maximum vessel diameter in PTS spinal cords (n = 11) was significantly greater (Fig. 2i; unpaired t-test, *p *< 0.0001) compared to controls (n = 15). MRW of perivascular microcavities, measured from the basal laminae of capillaries and postcapillary venules, ranged from 142 nm to 3 µm. Activated macrophages were frequently observed in the dilated perivascular spaces (Fig. 2d, f) as well as in the tissue surrounding syrinx cavities (Fig. 2g).

### Enlarged extracellular spaces

**Fig. 3:**
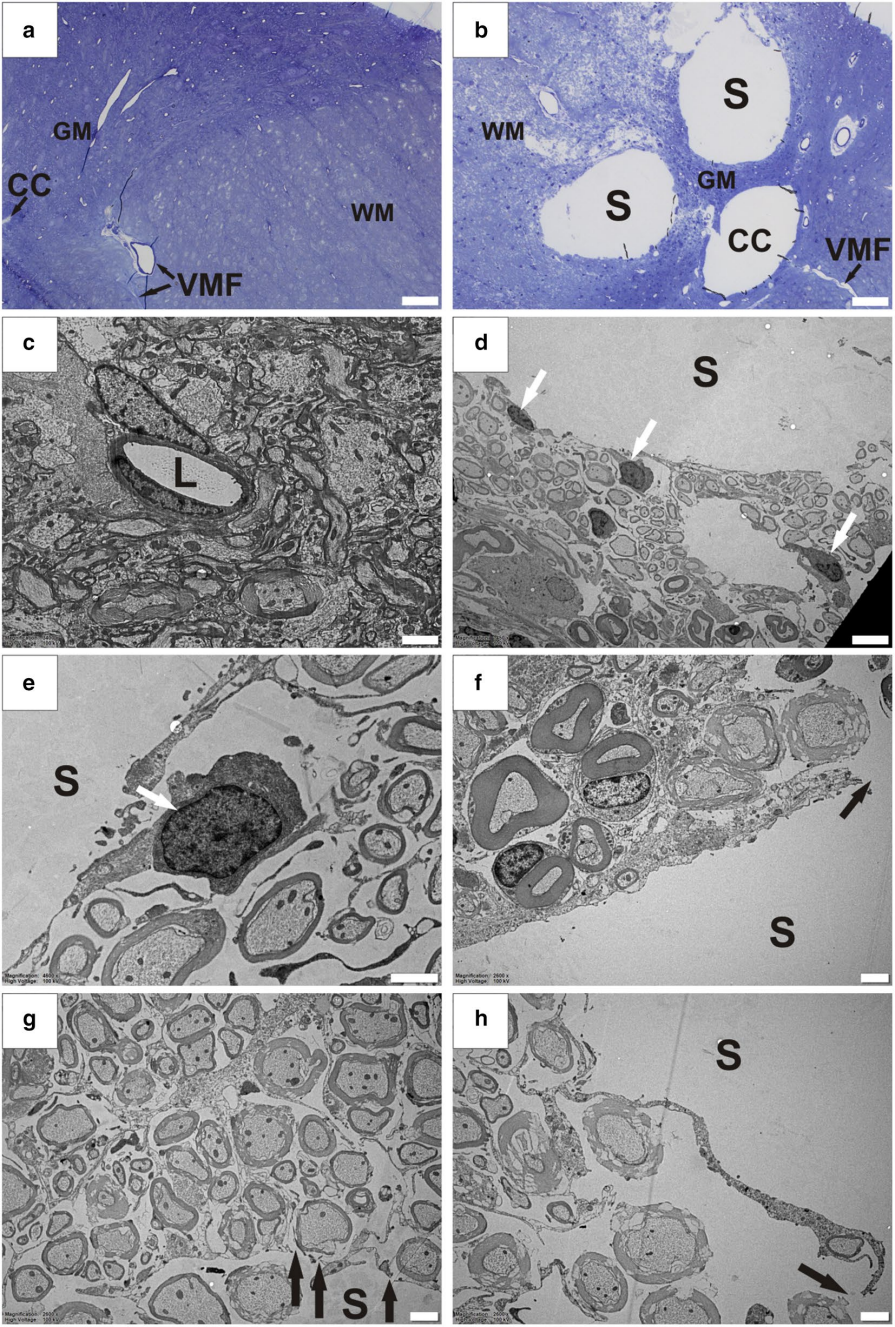
Enlarged extracellular spaces in the tissue surrounding syringomyelia cavities. Gross anatomical morphology of control (**a**) and PTS (**b**) tissue in semi-thin sections (750 nm) stained with methylene blue, displaying ventral median fissure and anterior penetrating vessel (VMF), central canal (CC), grey matter (GM), white matter (WM) and syrinx (S). The central canal in this PTS spinal cord (**b**) is dilated, but is not in direct communication with syrinx cavities. At the ultrastructural level, tissue appears normal in control animals (**c**). Enlarged extracellular spaces in the tissue surrounding syrinx cavities resembles edema and indicates increased water content in the spinal cord tissue (**d**). The syrinx border is lined by fragmented processes of astrocytes (euchromatic nuclei indicated by white arrows) and there appears to be a continuity (black arrows) between fluid in the cavity with the extracellular fluid and surrounding tissue (**d**–**h**). L, lumen. Magnification: ×3400 (**c**), ×1450 (**d**), ×4600 (**e**) ×2600 (**f**–**h**). Scale bars: 50 µm (**a**, **b**) 2  µm (**c**, **e**–**h**), 5 µm (**d**)

In contrast to healthy spinal cord tissue (Fig. 3a, c), the tissue surrounding syrinx cavities was characterized by loss of integrity and enlarged extracellular spaces (Fig. 3b, d–h). The loss of tissue integrity was associated with astroglial fragmentation and death, as well as loss of connective tissue. Axonal processes with disrupted myelin sheaths were frequently observed in the vicinity of syrinx cavities. This, however, may be an artefact of tissue processing and not characteristic of PTS.

### Astrocytes around syrinxes

Syrinx cavities were primarily lined by the processes of astrocytes, indicated by characteristic euchromatic nuclei (Fig. 3d, e; white arrows). However, in contrast to the continuous layers of cellular processes in healthy tissue, astrocytes lining syrinx cavities were often damaged and fragmented, resulting in discontinuities of the syrinx wall, and allowing communication between the cavity and the surrounding interstitial fluid (Fig. 3f–h; black arrows). The central canal was dilated when proximal to syrinx cavities (Fig. 3b). However, residual ependymal cells were not present around syrinx borders.

### Increased pinocytotic vesicles in endothelial cells

**Fig. 4:**
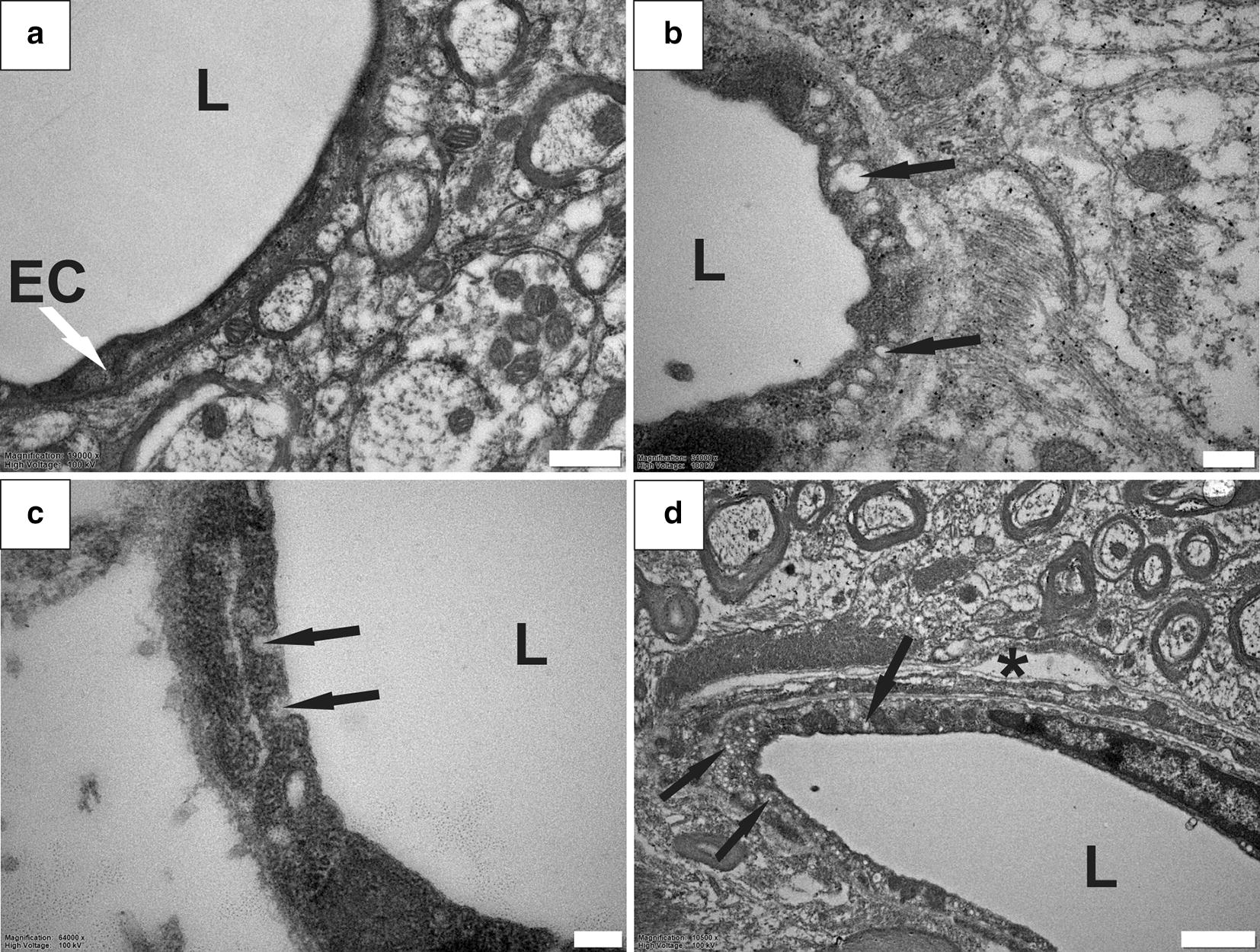
Abundant pinocytotic vesicles in endothelial cells in PTS. Blood vessels in healthy spinal cord tissue show a limited number of intracellular vesicles (**a**). In tissue from PTS animals, some blood vessels contained abundant electron-lucent vesicles indicated by black arrows (**b**–**d**). Intracellular vesicles fusing with the endothelial plasma membrane (**c**). Note the blood vessel in (**d**) also shows a microcavity in the perivascular region, suggesting that the two processes may be related. EC, endothelial cell; L, lumen; *, perivascular microcavity. Magnification: ×19,000 (**a**), ×34,000 (**b**), ×64,000 (**c**), ×10,500 (**d**). Scale bars: 0.5  µm (**a**), 0.2  µm (**b**), 0.1 µm (**c**), 1 µm (**d**)

Compared to blood vessels in healthy tissue (Fig. 4a), eight out of 24 (one in three) blood vessels assessed in the spinal cords of PTS animals contained an unusual abundance of intracellular vesicles (Fig. 4b–d). This was most noticeable in the capillary endothelium (Fig. 4b–d). Intracellular vesicles were electron-lucent and some appeared to be fusing with the endothelial plasma membrane (Fig. 4c). Abundant intracellular vesicles were observed in the endothelium of vessels that also exhibited perivascular microcavities (Fig. 4d).

### Altered BSCB

**Fig. 5:**
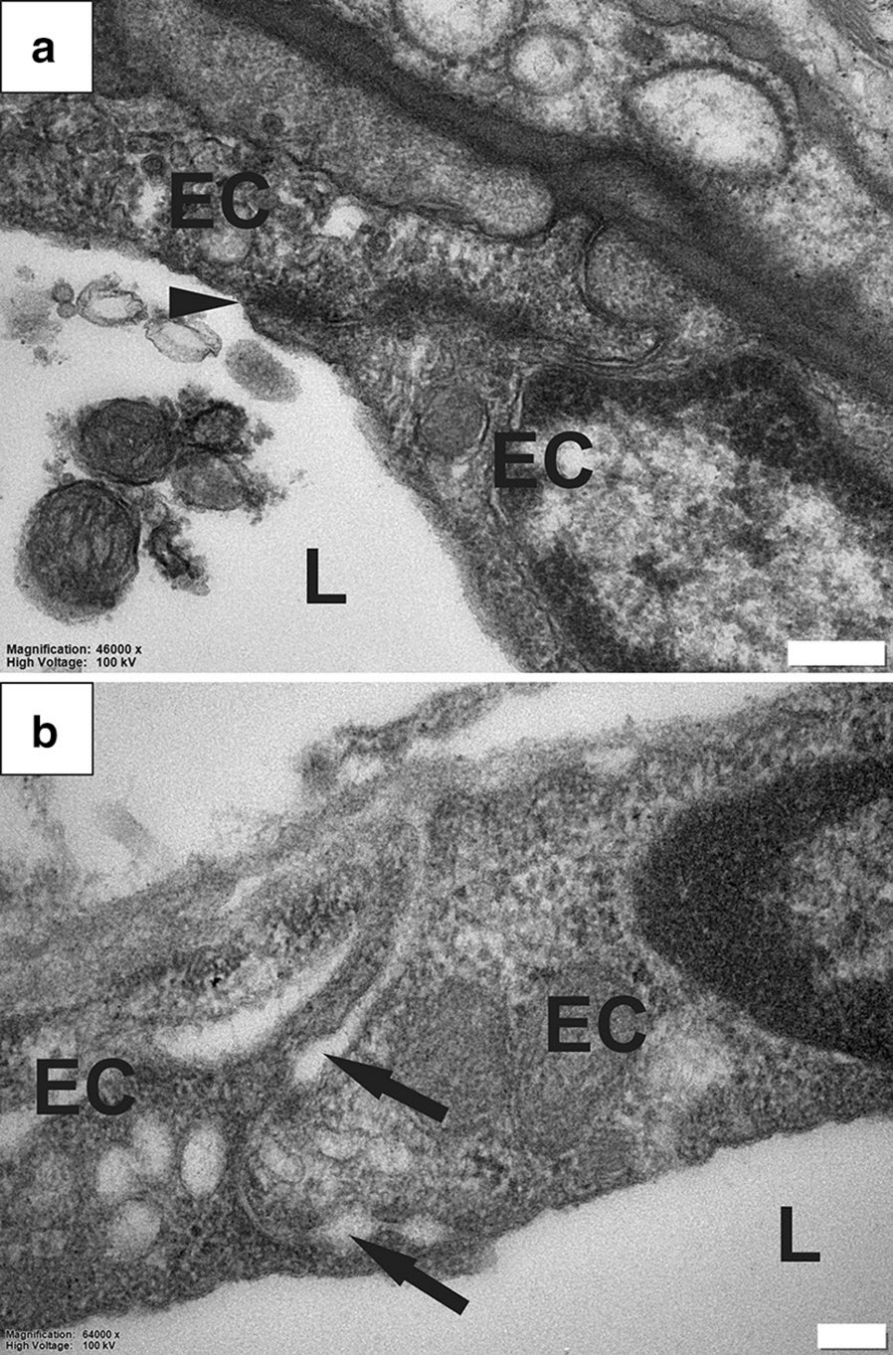
Abnormalities in the BSCB in PTS. Typical appearance of an occluding tight junction (black arrowhead) between capillary endothelial cells (EC), in the spinal cord of a healthy rat (**a**). Examples of abnormal capillaries in the rat spinal cord with PTS. The typical tight junctions between endothelial cells are not visible, and instead the intercellular junction is characterized by local swelling and dilation (black arrows). The intra- and extra-luminal spaces appear to be in continuity (**b**). L, lumen. Magnification: ×46,000 (**a**), ×64,000 (**b**). Scale bars: 0.2  µm (**a**), 0.1  µm (**b**)

The ultrastructure of endothelial tight junctions is a key feature of the blood–brain barrier (BBB) and BSCB. The characteristic electron-dense occluding junctional complexes [27] (Fig. 5a, arrow) were not clearly visible in some capillaries in the spinal cord tissue from PTS animals (Fig. 5b). Certain sections of the intercellular junctions appeared enlarged or swollen (black arrows) when the occluding junctional complexes were not visible. In these unusual blood vessels, the intraluminal and extraluminal compartments appeared to be in continuity.

## Discussion

This study investigated the ultrastructure of fluid flow pathways in a rat model of PTS. A detailed analysis of the spinal cord tissue revealed several abnormalities within perivascular spaces and the surrounding spinal cord tissue of animals with PTS compared with controls. In all animals, perivascular spaces were continuous with the extracellular spaces of the surrounding tissue and with the vascular wall basement membranes, consistent with previous data [21]. However, in animals with PTS, the perivascular spaces were strikingly enlarged with a concomitant loss of connective tissue, and often contained activated macrophages. Other abnormalities included widening of the extracellular spaces in the white and grey matter and loss of tissue integrity, consistent with severe parenchymal edema. Reactive astrogliosis, a pathological hallmark of spinal cord injury [28, 29] and syringomyelia [30], was not specifically identified in the vicinity of syrinx cavities in this study. However, the extensive tissue damage and loss at the injury level could, at least in part, be due to astrogliosis. Loss of tissue integrity was also accompanied by disrupted myelin adjacent to the syrinx cavity. Loose endothelial junctions (Fig. 5) and abundant pinocytotic vesicles (Fig. 4) at the level of capillary walls represent novel ultrastructural findings that may indicate altered BSCB function in this model of PTS. This is supported by previous findings using an excitotoxic model of PTS, which demonstrated that the BSCB is permeable to vascular tracers surrounding a syrinx [31]. Further, this is consistent with findings of increased vesicular transport and endothelial tight junctional opening adjacent to syrinxes in a leporine model of adhesive arachnoiditis [32]. Overall, these studies support the hypothesis that perivascular spaces play an important role in facilitating fluid flow in the spinal cord tissue, and their integrity is compromised in PTS. An impaired BSCB and increased vesicular transport may provide an additional route of fluid ingress that destabilizes normal volume regulation of the spinal cord.

The astrocyte-lined cavities (Fig. 3) and enlarged perivascular spaces (Fig. 2) reported in this study are consistent with earlier reports on the ultrastructure of human and rabbit syrinx cavities [14, 15, 32, 33]. Interestingly, enlarged perivascular spaces were also reported in hydrocephalus [34–36] and a number of cerebrovascular and systemic pathologies including small vessel disease [37, 38], moyamoya disease [39], ischemic and lacunar stroke [40], lupus erythematosus [41], and arteriosclerosis [42]. Despite frequent reports [34–42], the significance of enlarged perivascular spaces remains unclear. There is a possibility that blood vessels with enlarged perivascular spaces are initially involved in the process of syrinx formation, however there was no explicit evidence of enlarged perivascular spaces directly feeding into the cavities in this study.

In the present study, there appears to be a network–wide enlargement of the perivascular space in PTS (Fig. 2). Enlarged perivascular spaces are hypothesized to occur from perturbations to subarachnoid CSF flow, and may increase the fluid load of the cord [12]. Indeed, enlarged perivascular spaces have been observed in syringomyelia in human [43], and in animal models [16, 20]. Perivascular spaces exist within the functional complex of actrocytes, pericytes, endothelia and smooth muscle cells known as the neurovascular unit [44]. The coupling of perivascular flow to cerebral blood flow has been suggested in the brain to be involved in the homeostasis of the neurovascular unit by allowing metabolic waste clearance, regulated by astroglial flux [45]. Astrocytic endfeet that border perivascular spaces also maintain vessel tone through vasoactive metabolite release at vascular smooth muscle, known as neurovascular coupling. In this way, glial influence over CNS blood flow allows coordination of local energy demands [46]. Whether enlarged perivascular spaces play a role in neurovascular uncoupling, where neuroglial control over local vessel tone is lost, has yet to be illuminated. At the arteriolar level, it is possible that enlargement of the perivascular space may create a distance-based deficit, where vasoactive metabolites are diluted in the enlarged pool of fluid surrounding the tunica media. Indeed, this hypothesis would complement the theory that fluid loading of the spinal cord results from a mistiming of CSF and arterial pulse waves when the spinal subarachnoid space is obstructed [47, 48]. Here, vasoactive metabolites may be delayed in reaching smooth muscle across the enlarged perivascular space of parenchymal arterioles. The enlarged spaces seen around capillaries and venules (Fig. 2). may also relate to changes to endothelia (Fig. 4) and tight junctions (Fig. 5) found in this study. The microcavities may create a functional separation of astrocyte from endothelium. Astrocytes, as well as pericytes, are known to regulate and maintain the functional integrity of the BBB and BSCB [49–53]. The characteristic endothelial tight junctions of the BBB and BSCB may be altered if astrocytic signalling is delayed.

Another interesting finding of this study is the presence of ruptured myelin sheaths around axons adjacent to syrinxes, which has not been reported previously in syringomyelia, but is consistent with neurological deficits observed in animal models [54] and human patients [55, 56]. The death of oligodendrocytes and demyelination are common in neuroinflammatory conditions characterized by microglial activation and astrogliosis [57], supporting the notion that inflammation may contribute to the pathology of syringomyelia. The appearance of myelin lamellae separation in this study, however, may be an artefact of tissue fixation and not degradation secondary to oligodendrocyte death. Still, the extent of damage to myelin was more pronounced in PTS spinal cords compared to controls. Further investigation is required to determine whether this is a true characteristic of PTS pathology.

The use of electron microscopy affords unprecedented resolution of biological structures, however this method is labor intensive and precludes high-throughput analyses. This is reflected by the small sample size and single time point investigated in this study. Nevertheless, this approach proved useful for investigation of spinal cord anatomy at ultrahigh resolution. In animals with syringomyelia, this approach enabled the identification of previously unrecognised, physiologically important, anatomical changes, adding further knowledge to this complex neurological condition. Based on the electron micrographs acquired in this study, the direction of the pinocytotic vesicle transport could not be determined. Further studies are needed to investigate these processes and the integrity of the BSCB in this model of PTS, with the aid of CSF and vascular tracers.

## Conclusion

This study examined the ultrastructure of the spinal cord in an animal model of PTS. Abnormal changes to parenchyma, perivascular spaces and the BSCB may help to illuminate the pathological processes underlying this disease. If the tight regulatory coupling between glia and the vasculature is disturbed as it appears to be when perivascular spaces are enlarged, normal function of the BSCB may be altered. The findings of this study closely resemble pathological traits found in hydrocephalus and a number of neurovascular and neuroinflammatory conditions. Therefore, therapies developed for syringomyelia may have wider applications. Future studies should investigate the effect of ultrastructural changes on fluid flow in syringomyelia models using a combination of TEM and fluid tracers.

## Data Availability

The datasets supporting the conclusions of this article are available from the corresponding author on reasonable request.

## Abbreviations

BBB: blood brain barrier
BSCB: blood spinal cord barrier
CNS: central nervous system
CSF: cerebrospinal fluid
kDyn: kilodyne
MRW: maximum radial width
PTS: post-traumatic syringomyelia
TEM: transmission electron microscopy
